# Family-related non-abuse adverse life experiences occurring for adults diagnosed with eating disorders: a systematic review

**DOI:** 10.1186/s40337-020-00311-6

**Published:** 2020-07-22

**Authors:** Katie Grogan, Diarmuid MacGarry, Jessica Bramham, Mary Scriven, Caroline Maher, Amanda Fitzgerald

**Affiliations:** 1 School of Psychology, University College Dublin, Dublin, Ireland; 2 Elm Mount Unit, St. Vincent’s University Hospital, Dublin, Ireland

**Keywords:** Anorexia nervosa, Bulimia nervosa, Binge eating disorders, Eating disorders, Non-abuse adverse life experiences, Family adversity, Adults

## Abstract

**Background:**

Although previous reviews suggest a strong association between abuse and eating disorders, less is known about non-abuse adverse life experiences, such as parental mental illness or family discord, which occur frequently for this population. The aim of the current study was to identify family-related non-abuse adverse life experiences occurring for adults with eating disorders, and to establish whether they occur for people with anorexia nervosa, bulimia nervosa or binge-eating disorder more than the general population and other psychiatric populations.

**Method:**

A systematic review of studies focusing on family-related non-abuse adverse life experiences and eating disorders was conducted in accordance with PRISMA guidelines. The search string was applied to four electronic databases including Psycinfo, PubMed/Medline, CINAHL Plus and EMBASE.

**Results:**

Of the 26 studies selected for inclusion, six types of family-related non-abuse adverse life experiences were identified: adverse parenting style; family disharmony; loss of a family member, relative or close person; familial mental health issues; family comments about eating, or shape, weight and appearance; and family disruptions. Findings provided tentative evidence for eating disorder specific (i.e. parental demands and criticism) and non-specific (i.e. familial loss and family disruptions) non-abuse adversities, with findings also suggesting that those with bulimia nervosa and binge-eating disorder were more impacted by loss, family separations and negative parent-child interactions compared to those with anorexia nervosa.

**Conclusions:**

This review provides a clear synthesis of previous findings relating to family-related non-abuse adverse life experiences and eating disorders in adults. Implications for trauma-informed care in clinical practice were discussed (e.g. considering the impact of past life events, understanding the function of ED behaviours, reducing the risk of potential re-traumatisation).

## Plain English summary

There is evidence to suggest the significance of family factors beyond abuse in the lives of people with eating disorders. Yet, there have been few attempts in previous literature to demonstrate the importance of these family factors in eating disorder prognosis and recovery. This is the first systematic review completed on the topic of adversity that explores adverse life experiences beyond abuse in the context of eating disorders. Six types of family-related non-abuse adverse life experiences were identified: adverse parenting style; family disharmony; loss of a family member, relative or close person; familial mental health issues; family comments about eating, or shape, weight and appearance; and family disruptions. This study demonstrated that adversities occurring in the context of home and family environments are associated with having bulimia nervosa and binge-eating disorder to a greater extent than anorexia nervosa. These findings suggest that the course of anorexia nervosa may be less influenced by psychosocial factors such as family background issues. The paper discussed how the shared feature of bingeing among bulimia nervosa and binge eating disorder may occur as a reaction to family-related stress, or may have emerged as a coping mechanism for dealing with complex family backgrounds. This review provides evidence for the suitability and usefulness of trauma-informed approaches and recovery-oriented perspectives in clinical practice during the treatment of eating disorders. Avenues for future research have been clearly indicated in order to present more conclusive findings regarding specific family adversities.

## Review

Adversity, also known as ‘adverse life experiences’ (ALEs) within a mental health setting, includes any experiences or life events that have the potential to result in undesirable outcomes by disrupting normal functioning [61], and can be diverse in source, intensity and manifestation [47]. These experiences can be socially induced (e.g. child maltreatment, family discord), or they may occur naturally over time (e.g. parental loss, family illness). ALEs can occur at any point in a person’s life, but those occurring in the formative years (i.e. from age 0–18 years) are referred to as adverse childhood experiences (ACEs). Recent research has brought about increased awareness of the consistent relationship between exposure to ACEs or ALEs and long-term, negative mental health outcomes (e.g. [24, 33, 41, 58]).

Although the definition of ALEs is broad and encapsulates many diverse forms of adversity, research investigating ALEs in relation to eating disorders (EDs) has focused predominantly on childhood abuse, to the extent that various systematic reviews and meta-analyses have been conducted (e.g. [12, 43, 51]). These reviews collectively support the association between various forms of childhood abuse (i.e. sexual abuse, physical abuse, physical neglect, emotional abuse and emotional neglect) and bulimia nervosa (BN), binge eating disorder (BED), and to a lesser extent anorexia nervosa (AN). The inclusion of psychiatric control groups (i.e. participants with psychiatric disorders other than EDs) in these reviews allowed for the identification of disorder-specific (i.e. applying to people with EDs only) and non-specific (i.e. applying to people with psychiatric illnesses in general) results so that findings can be applied in clinical practice to better match treatment choices to specific clinical presentations [56]. Caslini et al. [12] also conducted ED subgroup comparisons (i.e. AN versus BN versus BED) to determine whether these three ED groups have underlying commonalities or differences, which in turn helps to establish the suitability of a ‘transdiagnostic approach’ to ED treatment [21, 23].

Although the association and impact of the relationship between childhood abuse and EDs appear to be firmly established, less research has examined the relationship between other non-abuse forms of ALEs and EDs. Non-abuse ALEs include any of the previously noted ALEs (e.g. parental loss, family discord etc.) but exclude abuse (i.e. sexual abuse, physical abuse, emotional abuse and neglect). These non-abuse ALEs, which are commonly family-based in nature, have sometimes been referred to as ‘cumulative adversity’ or ‘micro-traumas’ due to their negative impact over time, despite appearing less severe than abuse. The detrimental effects of these forms of trauma are further enhanced when the perpetrator is unaware of the impact of their actions, and moreover, if the victim feels ashamed when they cannot attribute their psychological distress to a major trauma or horrific event, leading to higher levels of emotional suffering and self-blame [13].

There are distinct reasons why we need to be better informed about non-abuse ALEs in relation to mental health outcomes. Firstly, non-abuse ALEs may occur more frequently than more extreme forms of adversity, such as abuse. For instance, Kessler et al. [33] completed surveys in nine countries (Belgium, France, Germany, Israel, Italy, Japan, The Netherlands, Spain and USA) to assess levels of childhood adversity. Results demonstrated that rates were higher for parental death during childhood (12.5%) compared to physical abuse (8.0%), sexual abuse (1.6%) or neglect (4.4%) among their sample of 51,945 adults, and that other non-abuse ALEs such as parental divorce (6.6%), family violence (6.5%) and parental mental health illness (6.2%) occurred in a significant proportion of cases. Secondly, there is evidence to suggest that just as abuse is associated with multiple psychiatric outcomes, so too are non-abuse ALEs. For instance, odd ratios (ORs) showed significant associations between DSM-IV disorders and abuse (i.e. physical abuse OR = 1.8; sexual abuse OR = 1.8; neglect OR = 1.5) as well as non-abuse ALES (e.g. parental mental illness OR = 2.0; parental substance misuse OR = 1.6; family violence OR = 1.6) [33]. Importantly, Kessler et al. [33] demonstrated that adversities associated with maladaptive family functioning, specifically (e.g. parental mental illness, parental substance misuse, family violence) had a greater impact on mental health disorders, compared with ‘other childhood adversities’ (e.g. serious physical illness).

There are clear clinical implications for adversity findings in the context of EDs. Mental health researchers and clinicians worldwide are now moving towards recovery centred approaches in working with people with various mental health diagnoses, which involves viewing recovery as a process, and not an end goal to be reached [29]. In line with the introduction of recovery-oriented principles in the care of people with mental health diagnoses in recent years, clinicians are encouraged to utilise a trauma-informed perspective in the management and treatment of various disorders [73]. Trauma-informed perspectives involve a consideration of the relevance of trauma and adversity in not only the development and maintenance (i.e. risk) of various mental health diagnoses, but also in terms of prognosis. In other words, instead of focusing on how adversity might impact on ED onset alone, knowledge about the role of adversity will also aid clinicians in their selection of appropriate interventions and treatments by generating a clearer clinical picture of the contextual factors contributing to client’s difficulties. Such trauma-informed work can help clinicians to facilitate a person to regain control and personal responsibility in working through their difficulties, resulting in resilience and improved recovery outcomes [73]. In this sense, such adversity research is not invested in exploring the causal relationship between certain adversities and later ED onset (i.e. risk), but instead to benefit patient prognosis by allowing clinicians to work from a more trauma-informed and recovery-oriented perspective.

The aim of the current study was to identify the various non-abuse ALEs occurring for adults with EDs by conducting a systematic review of the literature, with a particular focus on family-related non-abuse ALEs. This is the first systematic review examining adversity in the context of EDs that is not focused on abuse. The objective of this study was to provide information for clinicians regarding the potential family-related non-abuse ALEs which may impact individuals throughout their ED recovery. The four specific research questions were 1) What were the various family-related non-abuse ALEs reported to have occurred for adults with EDs? 2) What differences in these ALEs were reported by those with EDs compared to non-clinical samples (i.e. members of the general population without an ED diagnosis)? 3) What differences in these ALEs were reported by those with EDs compared to other psychiatric control groups? And 4) What differences in these ALEs were reported between the various ED subgroups (i.e. AN vs. BN vs. BED)?

## Method

### Study design

The current study was completed in accordance with Preferred Reporting Items for Systematic Reviews and Meta-Analysis (PRISMA [42];) guidelines. A protocol for this systematic literature review was registered with Prospero (protocol ID: CRD42019121905). Both qualitative and quantitative studies were included, and PICOTS parameters were used to assist in defining further inclusion and exclusion criteria in relation to study population, intervention/exposure, comparison, outcome, timing and setting (see Table 1).

**Table 1:** PICOTS parameters outlining inclusion/exclusion criteria for the database searches

Parameter	Inclusion	Exclusion
Population	Adults who report adverse life experiences in childhood or adulthood Adults with eating disorders including anorexia, bulimia, eating disorder not otherwise specified/ unspecified feeding or eating disorder, binge eating disorder Adults with obesity who also have a diagnosis of an eating disorder	Children (age < 18) Populations who do not have a diagnosed eating disorder Populations with obesity but no ED Populations not exposed to adverse/traumatic life experiences Animal studies
Intervention/Exposure	Family-related adverse/traumatic childhood experiences Family related adverse/traumatic adulthood experiences	Childhood abuse (including sexual abuse, physical abuse, emotional abuse or neglect) Adulthood abuse (including sexual abuse, physical abuse, emotional abuse or neglect) Health problems Social adversity (e.g. bullying at school)
Comparator	Healthy control participants Psychiatric control participants No comparison	
Outcome	No restriction on outcome Participants may or may not have recovered from their eating disorder	
Timing	No restriction on duration of negative life event Negative life event can be experienced in either childhood or adulthood	
Setting	No restriction on setting of intervention, but article must be published in English Articles must be peer reviewed	Articles published in language other than English Non peer-reviewed articles

### Search strategy

Four electronic databases were chosen for the literature search; Psycinfo, PubMed/Medline, CINAHL Plus and EMBASE. Searches were refined using filters which limited search results to English language only, peer-reviewed articles and articles published from the year 1980-present. Similar to previous adversity systematic reviews (e.g. [50, 64]), we chose to limit the search to research published from 1980 in order to ascertain relatively up-to-date findings. Furthermore, the DSM-III [1] was published in 1980, with earlier versions of the DSM having categorised EDs considerably differently compared to later versions. Each electronic database required slight adjustments to the search string depending on the database index terms, keywords and/or mesh terms (see Additional file 1 for sample search string). However, boolean searching, and methods for both broadening and narrowing the searches were utilized across all databases based on the two concepts being searched; EDs and ALEs. The search strategies for each of the four database searches were reviewed and checked by two librarians with expertise in systematic reviews.

### Study selection

**Fig. 1:**
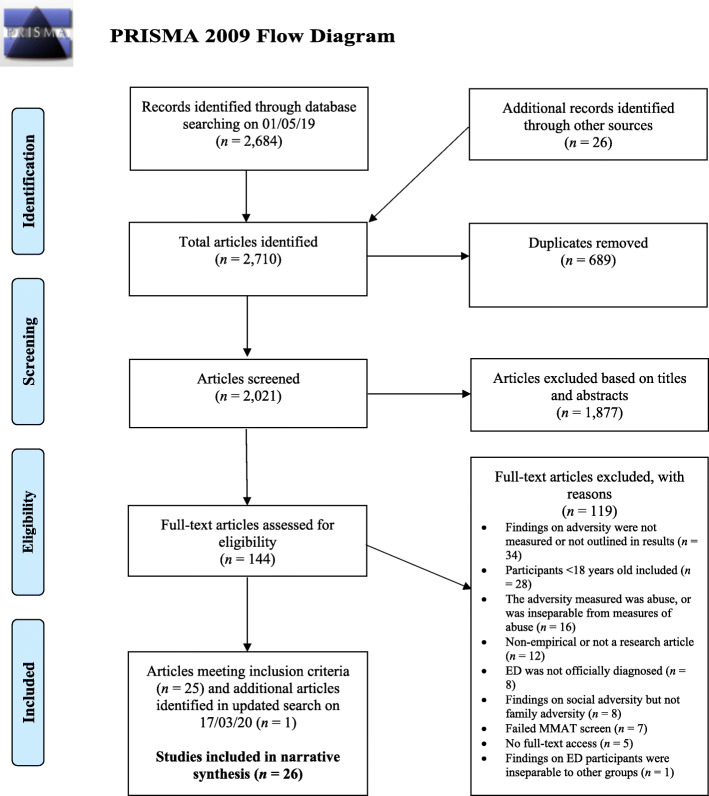
Flow chart of the study search and selection process

Database searches and importing of selected studies for screening took place on 1st May 2019. Figure 1 depicts the search and selection process which occurred for the current study. A total of 2684 studies were identified by applying the search string to the four databases, and an additional 26 studies were identified by scanning reference lists of included studies. This resulted in 2710 articles being imported for screening to Covidence [32], an online systematic review management software. This figure was reduced to 2021 once duplicates (*n* = 689) were removed. Titles and abstracts were screened by two independent authors (K.G and D.M.G), with an 88% agreement rate between authors. Disagreements between authors were resolved through discussion, and where consensus was not reached, a third author was consulted (A.F). A total of 1877 abstracts did not meet inclusion criteria, resulting in 144 studies remaining for full-text screening. Full-texts were obtained from various sources (i.e. multiple university libraries, Google Scholar and Library Genesis). For a minority of studies, full-text article download was unavailable, and so the authors were contacted in order to request access. If no response was received within 4 weeks, the article was deemed unavailable. Full-text screening was conducted by the same authors, with an agreement rate of 79% (*Kappa coefficient* = .58; moderate range), again with any conflicts being resolved via consensus or consultation with a third author. One hundred and twelve studies were excluded after this stage, with reasons provided in the flow diagram (see Fig. 1). An updated search was completed across databases once again on 17th March 2020, which led to one further study being identified which met inclusion criteria.

### Quality assessment

All remaining studies at this point (*n* = 33) were assessed using the Mixed Methods Appraisal Tools (MMAT) screening criteria [28] in order to critically appraise methodological quality. MMAT has been found to be an easy-to-use tool with moderate to perfect inter-rater reliability [49]. The two screening criteria were ‘Are there clear research questions?’ and ‘Do the collected data allow to address the research questions?’ The authors suggest that further appraisal might not be feasible or appropriate if answering ‘No’ or ‘Can’t tell’ to one or both of these screening questions. Seven studies failed to meet these screening criteria resulting in exclusion from the review. The methodological quality of the final set of studies for inclusion (*n* = 26) was assessed using MMAT criteria [28]. For the purpose of assessing studies included in the current literature review, the qualitative, quantitative non-randomised and quantitative descriptive tools were utilised and findings were reported (see Table 2).

**Table 2:** Description of included studies, quality ratings of study methodologies and information on study findings

Study information	Participant information			Quality	Details and findings reported							
Author (year) Country	Data source Total sample (age in years)	% female	Comparisons	MMAT	Measure of adversity	Findings						Abuse (y/n)
						Adversity domain						
						A1	A2	A3	A4	A5	A6	
A. Qualitative design												
1. Arthur-Cameselle et al. (2017) [2] USA	College sample *N* = 29 (*M* = 20.1; range = 18–24)	100%	Athletes with ED (A: *n* = 12; *M* = 20.5) **vs.** Non-athletes with EDs (NA: *n* = 17; *M* = 19.8). [EDs: AN (*n* = 17), BN (*n* = 3), BED (*n* = 1), AN+BN (*n* = 8)]	1.1 1.2 ● 1.3 ● 1.4 ● 1.5 ●	Semi-structured interview				✓	✓		Y
2. Reid et al. (2019) [60] UK	ED charity in Northern England *N* = 16 (Range = 19–58)	94%	No comparisons [EDs: not specified]	1.1 ● 1.2 ● 1.3 1.4 ● 1.5 ●	Adjusted version of Dan McAdams Life Story Interview			✓				Y
B. Descriptive/ non-comparative design												
3. Duarte & Pinto-Gouveia (2017) [20] Portugal	Treatment seekers from a university hospital *N* = 114 (*M* = 36.62; *SD* = 37.62; range = 20–63)	100%	No comparison [EDs: BED = 100%]	4.1 ● 4.2 ● 4.3 ● 4.4 4.5 ●	Shame Experiences Interview					✓		Y
4. Noordenbos et al. (2002) [46] The Netherlands	Dutch Foundation of AN and BN *N* = 41 (*M* = 34.3; *SD* = 7.6; range = 25–53)	100%	No comparison [EDs: AN = 44%, AN+BN = 41%, BN = 15%]	4.1 ● 4.2 ● 4.3 ○ 4.4 ● 4.5 ○	Self-report questionnaire		✓					Y
5. Sweetingham & Waller (2008) [68] UK	Specialist ED service *N* = 92 (*M* = 28.5; *SD* = 8.17; range = 18–58)	100%	No comparison [EDs: ANR = 13%, AN-BP = 8%, BN-P = 27%, BN-NP = 8%, EDNOS = 44%]	4.1 ● 4.2 ● 4.3 ● 4.4 4.5 ●	Experience of Shame Scale					✓		Y
C. Observational/ comparative design												
6. Boumann & Yates (1994) [5] USA	University hospital, ED clinic and advertisement in general population *N* = 50 (Range 18–43)	100%	BN (*n* = 25) **vs.** Control (*n* = 25)	3.1 ● 3.2 ● 3.3 ● 3.4 ○ 3.5 ●	Family History Research Diagnostic Criteria interview		✓		✓			N
7. Calam et al. (1990) [10] UK	Clinical practice, self-help groups, university and places of employment *N* = 380	100%	ED (*n* = 98) **vs.** Control (*n* = 242) [EDs: AN = 31%, BN/Hx AN = 35%, BNX = 34%]	3.1 ● 3.2 ● 3.3 ● 3.4 ● 3.5 ●	Parental Bonding Instrument	✓						N
8. Connan et al. (2007) [15] UK	Specialist inpatient unit, register, university advertisement *N* = 47 (Range = 18–45)	100%	AN (*n* = 18, *M* = 26.4, *SD* = 6.4) **vs.** R-AN (*n* = 13, *M* = 27.4, *SD* = 4.5) **vs.** Controls (*n* = 16, *M* = 27.5, *SD* = 4.6)	3.1 ● 3.2 ● 3.3 ● 3.4 ● 3.5 ●	Measure of Parental Style	✓						Y
9. Cuijpers et al. (1999) [16] The Netherlands	Netherlands Mental Health Survey and Incidence Study *N* = 7046	49%	ACOA (*n* = 586) **vs.** non-ACOA (*n* = 6460)	3.1 ● 3.2 ○ 3.3 ● 3.4 3.5 ●	Interview				✓			Y
10. Dalle Grave et al. (1996) [17, 18] Italy	Inpatient treatment unit *N* = 103	100%	ANR (*n* = 30, *M* = 21.9, *SD* = 6.0) **vs.** ANB (*n* = 12, *M* = 25.7, *SD* = 4.0) **vs.** BN (*n* = 17, *M* = 22.1, *SD* = 2.9) **vs.** BED (*n* = 30, *M* = 36.4, *SD* = 13.2) **vs.** Obese (*n* = 14 *M* = 40.5, *SD* = 13.5)	3.1 ● 3.2 ○ 3.3 ● 3.4 ○ 3.5 ●	Semi-structured Interview			✓				Y
11. Dalle Grave et al. (1996) [17, 18] Italy	ED treatment unit *N* = 238	100%	ANR (*n* = 30, *M* = 21.9, *SD* = 6.0) **vs.** ANB (*n* = 22, *M* = 24.9, *SD* = 4.2) **vs.** BN (*n* = 24, *M* = 22.3, *SD* = 2.7) **vs.** BED (*n* = 30, *M* = 36.4, *SD* = 13.2) **vs.** Sch. (*n* = 20, *M* = 33.9, *SD* = 7.1) **vs.** Controls (*n* = 112, *M* = 18.1 years, *SD* = 0.8 years)	3.1 ● 3.2 ○ 3.3 ● 3.4 ○ 3.5 ●	Semi-structured interview			✓				Y
12. Degortes et al. (2014) [19] Italy	Outpatient ED unit *N* = 214	un.	BED (*n* = 107; *M* = 31.1; *SD* = 11.1) **vs.** BN (*n* = 107; *M* = 25.4; *SD* = 5.6)	3.1 ● 3.2 ● 3.3 ● 3.4 ● 3.5 ●	Semi-structured interview		✓	✓			✓	Y
13. Gonçalves et al. (2016) [25] Portugal	Specialised ED treatment setting, other treatment settings and schools/ universities *N* = 180	100%	BN (*n* = 60, *M* = 21.52, *SD* = 4.86) **vs.** Controls (*n* = 60, *M =* 21.50, *SD* = 4.81) **vs.** PC (*n* = 60, *M* = 21.45 *SD* = 4.86)	3.1 ● 3.2 ● 3.3 ○ 3.4 ● 3.5 ●	Oxford Risk Factor Interview	✓	✓		✓	✓	✓	Y
14. Lehoux & Howe (2007) [36] Canada	Outpatient ED unit *N* = 80 (Range = 18–38)	100%	BN (*n* = 40, *M* = 25.13, *SD* = 5.26) **vs.** Sisters (*n* = 40, *M* = 26.32, *SD* = 5.25)	3.1 ● 3.2 ● 3.3 ● 3.4 ● 3.5 ●	Sibling Inventory of Differential Experience	✓						Y
15. Machado et al. (2014) [38] Portugal	Specialised ED treatment setting, other treatment settings and schools/ universities *N* = 240	100%	AN (*n* = 86, *M* = 20.02, *SD* = 4.49) **vs.** Controls (*n* = 86, *M =* 20.08, *SD* = 4.24) * AN (*n* = 68, *M =* 19.74, *SD* = 4.76) **vs.** PC (*n* = 68, *M* = 19.79, *SD* = 4.74)	3.1 ● 3.2 ● 3.3 ● 3.4 ● 3.5 ●	Oxford Risk Factor Interview		✓		✓	✓		Y
16. Mangweth et al. (1997) [39] Austria	University advertisement *N* = 85	0%	Austrian ED (*n* = 30, *M* = 25.7, *SD* = 4.8) **vs.** Austrian control (*n* = 30, *M* = 23.8, *SD* = 3.1) **vs.** American ED (*n* = 25, *M* = 21.2, *SD* = 3.3)	3.1 ● 3.2 ● 3.3 ● 3.4 3.5 ●	Semi-structured interview	✓	✓					Y
17. Manwaring et al. (2006) [40] USA	Advertisement in the community *N* = 155 (*M* = 31.17; *SD* = 5.73; range = 18–40	100%	Binge-first BED (*n* = 125, *M* = 30.70, *SD* = 5.83) **vs.** Diet-first BED (*n* = 30, *M* = 32.07, *SD* = 5.46)	3.1 ● 3.2 ● 3.3 ● 3.4 ● 3.5 ●	Oxford Risk Factor Interview			✓				Y
18. Monteleone et al. (2019) [44] Italy	ED centre *N* = 177	100%	ANR (*n* = 41, *M =* 25.45; *SD* = 8.02) **vs.** BP (*n* = 59; *M* = 27.14; *SD* = 9.78) **vs.** Control (*n* = 77, *M* = 25.58, *SD* = 2.31)	3.1 ● 3.2 ● 3.3 ● 3.4 ● 3.5 ●	Parental Bonding Instrument	✓						Y
19. Pike et al. (2006) [54] USA	Database and advertisement *N* = 431 (Range = 18–40)	100%	BED (*n* = 162, *M* = 30.8, *SD* = 5.8) **vs.** Control (*n* = 162, *M* = 30.0, *SD* = 5.6) *** BED (*n* = 107, *M* = 30.6, *SD* = 5.9) **vs.** PC (*n* = 107, *M* = 29.5, *SD* = 6.7)	3.1 ● 3.2 ● 3.3 ● 3.4 ● 3.5 ●	Oxford Risk Factor Interview			✓			✓	Y
20. Pike et al. (2008) [53] USA	Database and advertisement *N* = 150 (Range = 18–40)	100%	AN (*n* = 50, *M* = 26.70, *SD* = 6.23) **vs.** Control (*n* = 50, *M* = 26.56, *SD* = 5.51) **vs.** PC (*n* = 50, *M* = 27.02, *SD* = 6.05)	3.1 ● 3.2 ● 3.3 ● 3.4 ● 3.5 ●	Oxford Risk Factor Interview, Parental Bonding Instrument	✓	✓	✓	✓		✓	Y
21. Schmidt et al. (1993) [63] UK	ED treatment unit *N =* 203	99%	ANR (*n* = 64, *M* = 24.0, *SD* = 6.2) **vs.** ANB (*n* = 23, *M* = 24.7, *SD* = 6.9) **vs.** BN/Hx AN (*n* = 37, *M* = 23.6, *SD* = 5.3) **vs.** BN (*n* = 79, *M* = 24.4, *SD* = 5.4)	3.1 ● 3.2 ● 3.3 ● 3.4 ● 3.5 ●	Semi-structured interview	✓	✓	✓	✓		✓	Y
22. Striegel-Moore et al. (2005) [65] USA	Consumer database and advertisement *N* = 321 (Range = 18–40)	100%	BED (*n* = 107) **vs.** PC (*n* = 107) **vs.** Control (*n* = 107)	3.1 ● 3.2 ● 3.3 ● 3.4 ○ 3.5 ●	Oxford Risk Factor Interview	✓	✓		✓		✓	N
23. Swanson et al. (2010) [67] UK	Inpatient treatment facility and university campus *N* = 119 (Range = 18–48)	100%	AN (*n* = 43, *M =* 24.67, *SD* = 6.81) **vs.** Controls (*n* = 76, *M* = 20.53, *SD* = 5.1)	3.1 ● 3.2 ● 3.3 ● 3.4 ○ 3.5 ●	Parental Bonding Instrument	✓						N
24. Tagay et al. (2014) [69] Germany	Inpatient clinic and private practice *N* = 103 (*M* = 29.11; *SD* = 10.53; range = 18–68)	100%	AN (*n* = 52; *M* = 28.32; *SD* = 11.67) **vs.** BN (*n* = 51; *M* = 29.88; *SD* = 9.34)	3.1 ● 3.2 ● 3.3 ● 3.4 ● 3.5 ●	Essen Trauma Inventory			✓				Y
25. Wade et al. (2007) [72] Australia	Twin Registry *N* = 1056 (*M* = 35; *SD* = 2.11; range 28–40)	100%	AN = 23 **vs.** BN = 20 **vs.** MD = 186 **vs.** Control = 393	3.1 ● 3.2 ○ 3.3 ● 3.4 ● 3.5 ●	Family Life Events interview, Oxford Risk Factor Interview, Parental Bonding Instrument, Revised Moos Family Environment Scale	✓	✓			✓		N
26. Webster & Palmer (2000) [74] UK	ED services, psychiatric unit of general hospital, general medical practices *N* = 160 (Range = 18–49)	100%	AN (*n* = 28, *M* = 29) **vs.** BN (*n* = 32; *M* = 30) **vs.** AN+BN (*n* = 20; *M* = 30) **vs.** MD (*n* = 40; *M* = 34) **vs.** Control (*n* = 40, *M* = 34)	3.1 ● 3.2 ● 3.3 ● 3.4 ● 3.5 ●	Childhood Experience of Care and Abuse Interview	✓	✓					Y

A1 = Adverse parenting style; A2 = Family disharmony; A3 = Loss of a family member, relative or someone close; A4 = Familial mental health issues; A5 = Family comments about weight, eating or appearance; A6 = Family disruptions

MMAT *=* Mixed Methods Appraisal Tool; *N* = number of participants in total sample; *M* = mean age; *SD* = standard deviation; range = age range; *un. =* unknown; *n* = number of participants in sub-samples; ● = yes; ○ = no;  = can’t tell

*ED* Eating disorder, *AN* anorexia nervosa, *BN* bulimia nervosa, *BED* binge eating disorder, *AN+BN* mixed anorexia and bulimia, *ANR* AN restrictive subtype, *AN-BP* AN binging/ purging subtype, *BN-P* BN purging subtype, *BN-NP* BN non-purging subtype, *EDNOS* Eating Disorder Not Otherwise Specified, *BN/Hx AN* BN with a history of AN, *BNX* BN with no history of AN, *R-AN* Recovered AN, *ACOA* adult children of alcoholics, *Non-ACOA* non adult children of alcoholics, *ANB* AN binge eating/purging type, *sch.* schizophrenia, *PC* psychiatric controls, *BP* bingeing-purging, *MD* major depression

### Data extraction and synthesis

Data from selected studies were extracted by one author (K.G.) using a data extraction template form. A second author (D.M.G) completed double-extraction of 20% of papers for reliability purposes, demonstrating 87% agreement. Data extracted from each study included author, year, country, methodology/design, data source, percentage female, sample information, methodological quality score, measure of adversity and findings (Table 2). This table also took account of whether or not the studies included findings on abuse, which will not be discussed further in this review as this was not the focus of the research questions.

Owing to the heterogeneity of study designs, the inclusion of both qualitative and quantitative methods and the inclusion of diverse ED (AN, BN and BED) and control (psychiatric control and non-clinical controls^1^) populations in the final studies selected, meta-analysis was not appropriate for the data included in this review. Furthermore, there was a broad set of adversities included in this review, and various tools used to measure the adversity within the original studies. Narrative synthesis was therefore deemed the most appropriate form of analysis for these diverse data types so that nuances across studies can be captured adequately [62]. Narrative synthesis was conducted in line with Popay et al.’s [55] *‘Guidance on the conduct of narrative synthesis in systematic reviews’*. Specific tools and techniques outlined by the authors were utilised in the synthesis including *‘grouping and clusters’* and *‘tabulation’.* Research question 1 was addressed by identifying the various forms of non-abuse ALEs occurring for adults with EDs as reported in previous studies, and by grouping and clustering each variable into meaningful non-abuse ALE subheadings. Research questions 2, 3 and 4 were addressed using tabulation methods, whereby specific non-abuse ALE findings associated with ED versus controls, ED versus psychiatric controls, and ED subgroup comparisons were separated, described and synthesised. This allowed for a more succinct narrative synthesis of findings. Findings from the five non-comparative studies included rates of the adversity under investigation, with no between group comparisons made. Therefore, these studies were not referred to in the narrative synthesis, as they did not serve to answer research questions 2, 3 and 4, but findings on rates for these studies can be located in Additional file 2.

## Results

### Study characteristics

Twenty-six studies reported on family-related non-abuse ALEs in the ED literature (see Table 2), two of which were qualitative and the remaining were quantitative. The quantitative studies consisted of three descriptive/non-comparative studies and the remaining were observational/ comparative designs. The majority of studies were conducted in the UK (*n* = 7), followed by USA (*n* = 6), Italy (*n* = 4), Portugal (*n* = 3) and The Netherlands (*n* = 2), with only one study being conducted in each of Canada, Australia, Germany and Austria. The vast majority of these studies included female only samples (*n* = 21), however there was one study which included males only, one study whereby the gender of participants was not noted and three others which included 49, 94 and 99% female samples. The majority of the studies included mixed ED samples (*n* = 15), four studies included BED only samples, four studies included AN only samples, and three included BN only samples.

### Methodological quality of included studies

Twelve of the included studies met full criteria as assessed using MMAT, 10 studies met four of five criteria, and the remaining four studies obtained three of five criteria. No studies obtained fewer than three of five criteria. The limitations of the studies which obtained three of five criteria (*n* = 4) were identified during the synthesis of findings below so that results can be interpreted with caution by the reader.

### Findings on research question 1: family-related non-abuse ALEs occurring for adults with ED as reported in previous literature

Across the 26 studies, family-related non-abuse ALEs could be grouped under six broad subheadings: adverse parenting style; family disharmony; loss of a family member, relative or someone close; familial mental health issues; family comments about eating or weight, shape and appearance; and disruptions in family structure. Twenty-one of these 26 studies reported findings on abuse.

### Findings on research question 2: differences between people with EDs and non-clinical controls in relation to family-related non-abuse ALEs

#### Parenting style

Generally, ED groups demonstrated less favourable parent-child interactions during childhood compared to control groups, with evidence suggesting lack of care/ lack of affection/ increased parental indifference for people with AN [15, 44, 67], BN [10, 25, 44, 74] and mixed ED diagnoses [39, 74]. In contrast, Webster and Palmer [74] showed no differences between AN and control groups, and similarly Wade et al. [72] demonstrated no differences between AN, BN and control groups in terms of parental care. Two studies investigated these same parent variables by comparing adults with EDs to their siblings without EDs. Lehoux & Howe [36] showed that there were no differences in levels of parental affection between those with BN and sibling controls. Wade et al. [72] showed no differences between people with AN or BN and their unaffected twin comparisons for maternal care, but showed that people with BN experienced greater levels of paternal care than their unaffected twin comparisons.

Some studies demonstrated that those diagnosed with AN [15, 44, 72] and BN [36, 44] showed greater levels of parental over-control/overprotection when compared to non-clinical controls. However there was also evidence of the contrary, that there were no differences in levels of parental control for those with AN [10, 67, 74], BN [10, 25, 72, 74] or a mixed AN and BN group (i.e. AN/BN [74];) when compared to control participants.

In terms of other parenting factors, those with AN reported greater levels of paternal and maternal parenting problems [53] and higher parental demands [53]; people with BN (but not AN) reported greater levels of parental criticism [25, 72]; and people with general ED diagnoses reported lower quality relationships with fathers [39] when compared to controls. Wade et al. [72] reported no differences in relation to parental expectations between people with AN or BN and controls [72] whereas Gonçalves et al. [25] reported higher rates among those with BN compared to controls.

#### Family disharmony

Studies showed that rates of unresolved/ unaddressed family disagreements were higher among those with AN [38] and BN [25]; rates of witnessing inter-parental violence as well as perceived parental marital dissatisfaction were higher for those with general EDs [39]; and rates of family discord were higher for those with AN [53], BN and AN/BN (but not AN) [74], when compared to control participants. Boumann and Yates [5] showed that the odds of having divorced parents was 4.9 times higher for those with BN compared to controls. Machado et al. [38] failed to find differences between ED and control participants in terms of parental arguments, participant arguments with parents, arguments within the home and sibling rivalry. Moreover, Wade et al.’s [72] study which employed three different designs within their research (i.e. Design 1 = Diagnostic group comparisons; Design 2a = Monozygotic twin pairwise comparisons; Design 2b = Monozygotic twin case control) demonstrated conflicting findings with Design 2b suggesting that people with AN experience greater levels of parental conflict compared to controls and the other designs reporting no differences.

#### Loss

Pike et al. [54] demonstrated that people with BED reported more loss compared to controls whereas Pike et al. [53] reported that rates of loss did not differ between those with AN compared to controls.

#### Familial mental health issues

It was demonstrated that individuals with BN experienced higher rates of parental psychiatric disorder, major depression, personality disorders [5] and familial depression [25] compared to controls, but that rates of alcoholism [5, 25] or drug abuse [25] did not differ between the two groups. Machado et al. [38] also demonstrated no differences between their AN and control groups in relation to either family alcoholism or parental alcoholism. With a different design compared to other studies, Cuijper et al. [16] provided evidence that being an adult son of a parent with alcohol dependence was associated with ED psychopathology compared to adult sons of a parent without alcohol dependence, but that there were no differences between daughters of parents with or without alcohol dependence. However, these results should be interpreted with caution as authors measured parental alcohol dependence via self-report rather than using a standardised measure, and there was limited information on the demographics of study populations making it difficult to generalise findings. It was demonstrated that rates of familial EDs were higher for those with AN [38] and BN [25] compared to control families, however Pike et al. [53] showed that rates of familial AN and BN did not differ between their AN and control samples.

#### Family comments about eating, or weight, shape and appearance (i.e. physical appearance)

Machado et al. [38] demonstrated that there were no differences between their AN and control groups in terms of family comments about physical appearance. However, other studies demonstrated that individuals with AN [72] as well as those with BN [25, 72] experienced more parental comments about physical appearance when compared to a control group. Regarding comments about amounts eaten, increased rates of these sorts of comments were shown for those with AN [38, 72] as well as BN [72] when compared to control groups, but Gonçalves et al. [25] found no differences in family comments about eating between their participants with BN and controls.

#### Disruptions in family structure

Individuals with BED [54], but not those with AN [53], experienced more members leaving or joining the family structure (e.g. sibling being born, parents leaving home etc.) when compared to controls. There were no differences between individuals with BN and controls in relation to changes in parental figures [25].

#### Summary of ED vs. control findings

Greater levels of loss and family disruptions were experienced by those with BED compared to controls. There was evidence to suggest greater levels of family comments about physical appearance and eating for those with AN and BN compared to controls, but caution is warranted as not all studies supported this finding consistently. Although there are numerous studies suggesting a parental lack of care and over-control for people with EDs compared to controls, there is also a minority of studies suggesting no differences, implying a need for research to confirm these findings. Other parenting factors such as parental demands and criticisms appear to impact those with EDs compared to controls, but these findings must be replicated in order for them to be confirmed. Findings on family disharmony (i.e. family discord and disagreements) and familial mental health (i.e. familial ED and alcoholism) are inconclusive due to conflicting findings among included studies.

### Findings on research question 3: differences between people with EDs and psychiatric controls in relation to family-related non-abuse ALEs

#### Parenting style

Wade et al.’s [72] study demonstrated no differences between participants with AN or BN and major depression (MD) regarding parental care or control. Similarly, Gonçalves et al. [25] showed no differences between their participants with BN and psychiatric controls in terms of parental non-involvement. Webster and Palmer [74] demonstrated that there were no differences between four groups (AN, BN, AN/BN and depression) in terms of parental control, but that people with depression had higher levels of parental indifference and reported a lack of parental care compared to those with AN (but not BN or mixed AN/BN). In terms of other parental variables, it was demonstrated that people with AN and BN had higher levels of parental criticism than those with MD; that those with BN (but not AN) had higher levels of parental expectations when compared to psychiatric controls [25, 72], and that those with AN [53] and BED [65] reported higher levels of parental demands compared to psychiatric controls.

#### Family disharmony

Family discord was shown to be greater for those with AN [53] and BED [65] as compared to other psychiatric disorders. Similarly, higher rates of unresolved/ unaddressed family disagreements was reported for those with BN compared to psychiatric controls [25] and a trend towards a significant difference was demonstrated for those with AN compared to psychiatric controls [38]. In contrast, other studies reported no differences between those with ED versus psychiatric controls in terms of family discord (BN = AN/BN = AN = depression [74];), arguments with parents [38], sibling rivalry [38] and parental conflict [72].

#### Loss

No differences in loss were established between people with BED [54] or AN [53] compared to psychiatric controls. Across two other studies, rates of loss of a family member were reported at 17% for people with BED compared to 0–9% for people with obesity and schizophrenia, as well as AN-restrictive, AN-binge/purge and BN [17, 18]. However, the authors commented that values were too low to conduct statistical analysis and so these findings were not tested for statistical significance. It also must be noted that the methodological quality of these studies was weakened due to the measures used and a lack of consideration of confounding variables, as assessed using MMAT criteria.

#### Familial mental health issues

It was demonstrated that individuals with AN [38] and BN [25] had higher rates of familial ED compared to psychiatric controls, however Pike et al. [53] reported that rates of familial AN and BN did not differ between their AN and psychiatric control samples. Pike et al.’s [53] study showed that parental mood and substance disorder were experienced by those with AN and psychiatric controls combined more than controls, but that there were no differences between the AN and psychiatric control groups.

#### Family comments about eating, or weight, shape and appearance (i.e. physical appearance)

Machado et al. [38] demonstrated no differences between their AN and psychiatric control groups in terms of family comments about physical appearance or eating. However, it was demonstrated that individuals with BN experienced more comments about physical appearance [25, 72] and that people with both BN and AN experienced more comments about eating [72] when compared to psychiatric comparison groups.

#### Disruptions in family structure

Individuals with BED [54], but not those with AN [53], experienced more members leaving or joining the family structure compared to psychiatric controls. Striegel-Moore et al. [65] demonstrated that a mixed group of individuals with BED and psychiatric controls together reported higher ratings of parental separation when compared with controls, but no differences were shown between BED and psychiatric control groups. There were no differences between individuals with BN and psychiatric controls in relation to changes in parental figures [25].

#### Summary of ED versus psychiatric control findings

There is evidence to suggest higher levels of adverse parenting styles such as parental demands and criticism among people with EDs compared to psychiatric controls. Findings also suggest that people with BED experienced more members leaving or joining family structure compared to psychiatric controls. Further replication of these findings is required before drawing any strong conclusions. Findings on family disharmony are inconclusive, with some studies suggesting that this adversity is reported by those with EDs more than those with psychiatric controls, particularly for family dissonance (i.e. discord and disagreements), but there were also findings to suggest no differences between the groups. There was some evidence that family comments about physical appearance and comments about eating are reported more for those with BN compared to psychiatric controls, but there were conflicting findings regarding whether this adversity was experienced by those with AN more than psychiatric controls. Loss and family mental health issues were not experienced by those with EDs more than psychiatric control groups. There was some evidence to suggest that family mental health issues might be associated with psychiatric disorders in general, but not ED specific. Parental care and parental control findings mostly suggest no differences between ED and psychiatric control groups, with some evidence that parental indifference might be more significant to those with depression than AN.

### Findings on research question 4: differences among ED subgroups in relation to family-related non-abuse ALEs

#### Parenting style

Studies on parenting style consistently demonstrated that people with BN experience poorer child-parent interactions compared to those with AN. Webster and Palmer [74] showed that people with BN reported increased levels of parental indifference and a greater lack of care compared to their AN counterparts, but the groups did not differ in terms of control (over- or under-control). Similarly, Schmidt [63] assessed differences between the childhood experiences of four groups of ED participants including restricting AN, bulimic AN, BN with a history of anorexia and normal-weight BN, and demonstrated that normal-weight BN patients had experienced higher levels of parental indifference and over-control (but not under-control) than the other three groups. Monteleone et al. [44] demonstrated no differences between their AN restricting and bingeing-purging (BP) subgroups in relation to parental care or control, however it must be noted that the BP group consisted of people with both AN and BN diagnoses. Moreover, Connan [15] demonstrated no differences in parental indifference between active versus recovered AN groups, but because rates were higher in both these groups compared to controls, it can be concluded that these negative parental styles may occur for people with AN regardless of whether they recover or not.

#### Family disharmony

Findings showed that there were no differences in rates of family conflicts between a BED and BN group [19] and there were no differences in rates of family discord between those with AN and BN [74]. However, Schmidt et al. [63] showed that a normal-weight BN group reported a trend towards higher levels of intrafamilial discord compared to the restricting AN, bulimic AN or BN with a history of anorexia groups [63].

#### Loss

In terms of ED subgroup comparisons, loss was assessed as a precipitating factor in two of the studies, with Degortes et al. [19] having demonstrated that participants with BED reported more bereavements in the 6 months preceding ED onset compared to BN comparisons, and Manwaring et al. [40] having shown that a binge-first (i.e. bingeing symptoms began before dieting) group of BED participants were more likely to report having lost someone close to them in the year preceding ED onset when compared to a diet-first group (i.e. dieting symptoms began before bingeing). These studies considered the temporal sequencing of loss and ED onset, with findings suggesting that loss preceded bingeing behaviours, and therefore presents itself as a likely risk factor for the later onset of an ED, specifically BED. When comparing samples of those with AN and BN, Tagay et al. [69] demonstrated no statistical differences in relation to number of deaths experienced, however they reported that death of a close person or family member was considered the most traumatic form of life event by both groups, over-and-beyond other forms of adverse experiences including abuse/assault, imprisonment, natural disasters or having been involved in accidents such as a fire.

#### Parental mental health issues

Schmidt et al. [63] demonstrated that there were no differences between ED subgroups (i.e. restricting AN, bulimic AN, BN with a history of anorexia and BN) in relation to the frequency of maternal or paternal mental health difficulties.

#### Family comments about eating, or weight, shape and appearance (i.e. physical appearance)

None of the original studies included in this review conducted analyses on ED subgroup differences for this adversity subtype and so no findings can be reported here.

#### Disruptions in family structure

ED subgroup comparisons showed that individuals with BED experienced more separation from family members in the 6 months preceding ED onset than those with BN [19]. Schmidt et al. [63] analysed comparisons in four ED subgroups (i.e. restricting AN, bulimic AN, BN with a history of anorexia and BN) and showed that the three BN groups had 3 or more total changes in family arrangements (e.g. boarding school, adoption, parental separation, parental death and other) compared to the AN-restricting group, with parental separation being the most commonly reported reason for these changes.

#### Summary of ED subgroup findings

There was evidence to suggest that loss of someone close is linked to the later onset of bingeing behaviour/ BED and therefore might pose an ED risk, due to the consideration of temporal sequencing in the studies which assessed their relationship. Similarly, findings on separation of family members and parental separation also demonstrated that this adversity is reported more often for those with BED than BN, and more often for those with BN than AN, suggesting that perhaps the bingeing behaviours involved in BN and BED might result from people’s attempt to cope with these specific family adversities. Findings suggested that those with BN experience more negative parent-child interactions compared to those with AN in the form of parental indifference, in particular. Family disharmony or parental mental health issues did not appear to be associated with any particular ED subgroup. No ED subgroup analyses were conducted on family comments about eating or physical appearance and so no conclusions can be draw about this variable in terms of ED subgroup specificity.

## Discussion

### Summary of research aims and findings

The aim of the current systematic review was to identify the various forms of family-related non-abuse ALEs reported to have occurred for adults diagnosed with EDs. The four specific research questions were 1) What were the various family-related non-abuse ALEs reported to have occurred for adults with EDs? 2) What differences in these ALEs were reported by those with EDs compared to non-clinical samples? 3) What differences in these ALEs were reported by those with EDs compared to other psychiatric control groups? And 4) What differences in these ALEs were reported between the various ED subgroups (i.e. AN vs. BN vs. BED)? Six types of family-related non-abuse ALEs were reported in the ED literature, which included adverse parenting style; family disharmony; loss of a family member, relative or close person; familial mental health issues; family comments about eating or physical appearance; and disruptions in family structure. This is the first systematic review in the ED literature to identify adversities other than abuse which may impact people with EDs. Findings on these adversities will be summarised and discussed as per the remaining research questions.

### Differences between ED versus control groups

There was evidence to suggest that experiences of loss [54], family disruptions [54] and family comments about amounts eaten [38, 72] occurred more often for people with EDs compared to control participants. However, it must be noted that there was more consistent support for these findings for those with BED and BN compared to AN, and that replication studies are necessary in order to confirm these findings. However, these findings were not surprising considering the association that has been established between ALEs and various mental health issues (e.g. [24, 33, 41, 58]), suggesting a potential role of family-related non-abuse ALEs in the trajectory of EDs. Findings were more inconclusive for other variables such as family disharmony, familial mental health and parenting style. Studies investigating these variables were limited and conflicting findings were demonstrated among included studies, suggesting a need for further research on these adversities.

### Differences between ED versus psychiatric control groups

There was evidence that adverse parenting styles in the forms of parental demands and criticism [53, 65, 72] occur for those with EDs more than psychiatric controls; that people with BN [25, 72] but not those with AN [38, 72] experienced more parental comments about weight when compared to psychiatric controls; and that those with BED [54] but not AN [53] experienced more family disruptions in the form of family members leaving or joining the family structure compared to psychiatric controls. Replication studies are required to provide confirmation of these findings, though they provide tentative evidence of ED-specific family adversities. Again, these findings might suggest a relationship between family problems with BN and BED over-and-beyond AN.

Findings on loss were consistent, demonstrating no differences in rates of loss between those with AN or BED and psychiatric control participants [53, 54], suggesting that loss may be a non-specific adversity experienced by people with psychiatric difficulties in general compared to the general population. In terms of parental mental health issues, it appeared that results were varied depending on the type of mental health issues assessed. There was evidence to suggest that whereas familial mood, substance disorders and alcoholism might be general risk factors for various psychiatric illnesses [38, 53], parental EDs is associated with offspring ED but not other offspring psychiatric disorders [25, 38]. This finding is easily understood considering the “unequivocal evidence” of heritability of EDs, as suggested by Klump, Bulik, Kaye, Treasure & Tyson ([34], p.97), but it must be noted that Pike et al.’s [53] study failed to support this finding. However, Pike et al. [53] note that child-reporting of familial psychiatric illness might not fully capture true rates of the disorders that might exist within families. Findings on family disharmony (i.e. family discord and unresolved disagreements) were conflicting, resulting in an inability to draw conclusions on these variables.

### Differences between ED subtypes

There was evidence to suggest that loss of someone close and separation of family members are linked to the later onset of bingeing behaviour and BED when compared to dieting behaviours or BN [19, 40]; that separations within families were reported more often for those with BED than BN [19], and more for those with BN than AN [63]; and that those with BN experienced more negative parent-child interactions compared to those with AN, particularly parental indifference [63, 74]. This again suggests that these family adversities are significant in the lives of those with BED and BN to a greater extent than people with AN. Finally, findings on family disharmony and parental mental health difficulties suggested that these variables were not specific to any ED subtype. Although the distinction of ED subtype differences is useful for clinical purposes, research confirming no differences between ED subtypes across multiple variables supports the transdiagnostic approach to working with EDs due to apparent indistinct features across ED subtypes [21, 23].

### Considerations for the interpretation of results

Regarding the findings implying elevated rates of critical comments about physical appearance and eating for people with EDs, it must be considered that personal factors might also influence perception and subsequent reporting. For instance, research has suggested that people with EDs overvalue their body weight and shape [11, 22] and that people with EDs are prone to attentional biases to disorder salient stimuli (e.g. body size, food) that are perceived as potential threats [3]. This could impact recall bias whereby an ambiguous cue such as someone commenting on appearance might be interpreted in a more negative light due to its perceived threat for people with EDs. Alternatively, another consideration is that those with EDs may be accessing treatment that is oriented around increasing food intake and these comments are therefore likely to be elevated in such a setting. However, regardless of the purpose of the comments, previous research has demonstrated that such comments have been linked to adverse effects such as poorer self-esteem, lower perceived social support and larger perceived body size [70]. Similarly, caution must be advised in interpreting the finding that adverse parental styles were experienced by those with BN more than those with AN. Previous research has suggested that BN is associated with increased interpersonal sensitivity, including criticism and rejection sensitivities [26], so it must also be considered that increased reporting of these parenting styles might have been influenced by child perspectives and behaviours resulting from this increased sensitivity or poor attachment. This results in a difficulty in drawing conclusions on such results as the definition of ALEs suggests that these experiences be external and verifiable, and not internal or psychological [52, 57, 64].

A common finding evident across the research questions addressed was that these differences in family-related non-abuse ALEs were more prevalent for people with BN and BED than AN. It is therefore likely that the inclusion of AN groups in addition to other ED groups in studies assessing these questions in this current review may have impacted on or led to insignificant findings with regard to the two research questions relating to ED versus control and psychiatric control differences. Despite this, we can make the following inferences from these findings: that AN is less influenced by familial factors and that the trajectory of AN may be less connected to family environment of childhood upbringing when compared to BN or BED. This may provide an explanation for why family therapies demonstrate better efficacy for people with AN compared to other EDs due to more positive and intact family relations [31, 37]. Furthermore, the function of self-starvation, one of the hallmark symptoms of AN, has been associated with evading hurtful feelings, relational problems and high expectations in previous research [8, 48]. Starvation also leads to impairments of cognitive functioning, including memory and attention, for people with AN who are currently ill as well as weight-restored [45]. Therefore it must be considered that the lack of significant findings for the AN group in relation to non-abuse ALEs might reflect a buffering of such experiences as a consequence of functional avoidance of hurtful feelings or cognitive impairment impacting the accuracy of reporting. Despite this, this finding is in line with previous research which suggested that genetic influences are more significant for AN compared to BN [71]. Various authors have formed similar conclusions that individuals with BN and BED might experience more family-related ALEs compared to those with AN (e.g. [63, 74]), and similarly, findings on abuse suggested a clear association between abuse with both BN and BED, but to a lesser extent with AN [12]. The common symptom of bingeing for BN and BED might explain this association as it has been previously described as a manifestation of greater reactivity to stress [27] and is positively related to poorer interpersonal problem solving skills [66], suggesting that bingeing may come about as a reaction to family-related problems or as a coping mechanism for dealing with such situations. However, careful consideration is warranted in deciding whether to focus on such family problems during treatment, as a focus on these can be counterproductive to recovery, especially when treatment involves engaging the family.

### Implication of findings

There are a number of clinical implications stemming from the findings of this study, application of which would result in clinicians working from a trauma-informed perspective in line with recovery-oriented practice [7, 29, 73]. First, considering the relevance of adversity in the lives of people with EDs, clinicians may wish to use a life events checklist in order to assess for the presence of these adversities in the lives of their clients. For instance, if a person has experienced loss of a parent at a young age, experiential trauma-focussed work in combination with, prior to or subsequent to ED treatment may be effective. The order of treatment focus should be decided together with the client, in line with best practice recovery-oriented guidelines [14]. Second, according to the self-medication hypothesis, eating behaviours may serve a function for managing or avoiding trauma-related memories or cues [6]. Therefore, any attempts at removing or lessening such behaviours should take account of the function they serve so that people with EDs are not left without an alternative means to cope. Third, in relation to increased reporting of comments about weight or appearance, clinicians must take extra care of the language and choice of wording so as to minimise the risk of inadvertent re-traumatisation. Other treatment actions that should be carefully considered to avoid re-traumatisation include routine weighing and medical rescue interventions deemed aggressive and coercive, such as force feeding [7]. In summary, this trauma-informed approach to ED care should result in (i) a better understanding of the context of client’s difficulties, (ii) a better selection of appropriate treatment types and onward referral if necessary, and (ii) a reduction of the risk of accidental re-traumatisation by service-providers [9].

Finally, findings from this review demonstrated that certain distinctions may exist between the three disorders (particularly for BN and BED versus AN), suggesting the need for research to investigate these disorders separately to allow for the identification of disorder-specific findings. This is not necessarily to say that this research cautions against the use of a transdiagnostic approach to ED treatment which has been proposed by many experts [22], but that at least from a research perspective, empirical findings relating to subtype similarities and differences can assist with identifying features of the three disorders which may be addressed from transdiagnostic perspectives versus those which are specific to an ED subtype. For instance, previous research has demonstrated that not all processes identified in the transdiagnostic CBT-E model operate in the same manner across ED subtypes, but that the model provides guidance for clinicians on a range of factors which may be important in the maintenance of any ED subtype [35]. Lampard et al. [35] conclude that an understanding of both transdiagnostic mechanisms and mechanisms specific to ED subtypes can inform clinical practice.

### Quality of included studies

Included studies demonstrated good to excellent methodological quality as rated by MMAT, however studies were not without their limitations. All adversities were measured via retrospective self-report (i.e. standardised questionnaires, interviews etc.). Negative recall bias might skew results whereby individuals who are suffering with mental health issues may have a high tendency for threat detection [2] as well as a subjectively negative report of past experiences [4, 59]. Other issues regarding the methodologically weaker studies were mentioned previously, which include a lack of information on study demographics, small sample sizes, and a lack of consideration for confounding variables.

### Strengths, limitations and future recommendations

This review was conducted in line with best practice guidelines including PRISMA [42] and Cochrane [62]. The study aimed to minimise bias through the use of multiple reviewers during the screening, extraction and analysis phases, with evidence of moderate to high inter-rater reliability throughout. This review had a broad focus which was beneficial in terms of providing a representative view of the current literature base on a topic of adversity that has not been systematically reviewed in the ED context to date.

However, the studies included were highly heterogeneous due to the inclusion of diverse research designs, measures of adversity and ED populations. Future research could aim to use meta-analytical approaches by narrowing the scope of the research question to focus on distinct forms of family-related non-abuse ALEs (e.g. loss of family member). This might allow for the inclusion of further relevant studies which were not captured in the current search due to the broad research focus. Meta-analytical approaches would also help to determine whether the familial adversity is related to BN and BED more strongly than AN using statistical means. In terms of taxonomy, this review established correlates of EDs important for treatment considerations rather than risk, due to the lack of information on timing. As suggested by Jacobi et al. [30], reviews which aim to identify risk factors would benefit from establishing the interaction between biological and psychosocial factors, ideally through the utilisation of longitudinal design in place of retrospective cross-sectional designs. Furthermore, the use of ecological momentary assessment methods or other qualitative approaches would help reduce recall bias in the reporting of adversity, or similarly the use of informant reporting of events (e.g. sibling report). Literature published from 1980 onwards was reviewed in the current article, meaning that some relevant research published before this date may have been missed. The search was also restricted to articles in the English language only, which represents a potential selection bias as some authors may have published relevant findings which were not available for inclusion in this review. However, as the majority of studies appeared to be undertaken in the US and UK, we estimate that this risk of bias would be low. Furthermore, as DSM criteria for EDs have changed throughout the years, there may be issues relating to the classification of ED subtypes inherent in this research. As can be seen from the findings reported, older studies use terminology to describe EDs that do not exist today, and may result in an overlap of symptom presentations across ED subtypes. As the classification of mental disorders is ever-changing, there is strong encouragement through the research framework, Research Domain Criteria (RDoC), that future research should focus on symptoms rather than the categorisation of symptoms in investigating mental health disorders to overcome this issue. Finally, future research should investigate adversities occurring in those under age 18, which was beyond the scope of the current review.

### Summary and conclusions

Overall, these findings help us understand the role of ALEs, above and beyond abuse, in the overall trajectory of EDs. This review confirmed, as hypothesised, that adults with EDs are subject to higher rates of various forms of family-related non-abuse ALEs compared to non-clinical samples, with findings on loss and disruptions of family structure providing most compelling evidence. The review identified potential ED-specific ALEs, such as parental demands and parental criticism, which may occur more frequently when compared to other psychiatric groups. ED subgroup findings suggest that those with BN and BED in particular, may experience a greater level of family-related non-abuse ALEs, such as loss, family separation and negative parent-child interactions compared to those with AN. Collectively, findings suggested that AN may be less influenced by psychosocial factors connected to family environment during childhood compared to BN and BED [63, 74]. These findings are important to consider for clinicians working from trauma-informed and recovery-oriented approaches in ED treatment, as they will help to form a clearer picture of client’s difficulties and targets for intervention, which will benefit recovery outcomes.

## Supplementary information

**Additional file 1.** Sample search strategy.

**Additional file 2.** Tabulation of results addressing research questions 2, 3 and 4.

## Data Availability

Data sharing is not applicable to this article as no new data were generated or analysed during this study.

## Abbreviations

ACE: Adverse childhood experiences
ALE: Adverse life experiences
AN: Anorexia Nervosa
BED: Binge-eating disorder
BN: Bulimia Nervosa
BP: Bingeing-purging
DSM: Diagnostic and Statistical Manual of Mental Disorders
ED: Eating disorder
MD: Major depression
MMAT: Mixed Methods Appraisal Tools
OR: Odd ratio
PICOTS: Population, Intervention, Comparators, Outcome/exposure, Timing, Setting
PRISMA: Preferred Reporting Items for Systematic Review and Meta-Analyses
RDoC: Research Domain Criteria

## Footnotes

1 From here on out, the term ‘controls’ will refer to non-clinical participants whereas ‘psychiatric controls’ will refer to participants who do not have ED diagnoses, but do have other psychiatric diagnoses. In some instances, ‘control participants’ include non-clinical siblings of people with EDs, but this will be clearly outlined when discussed.
