# Supplementary material for: Family-related non-abuse adverse life experiences occurring for adults diagnosed with eating disorders: a systematic review
Source: J Eat Disord. 2020 Jul 22;8:36. doi: 10.1186/s40337-020-00311-6 (PMC7374817; doi:10.1186/s40337-020-00311-6)
Supplement: Supplementary file 1 — Additional file 1. Sample search strategy. [file 40337_2020_311_MOESM1_ESM.docx]

**Additional file 1: Sample search strategy**

**Table 1:** **Search strategy for Psychinfo as on 01/05/19 yielding 1,076 results**

|  | **Boolean operator** | **Term** | **Type** |
| --- | --- | --- | --- |
| ***Concept 1: Eating disorder*** |  | MJMAINSUBJECT.EXACT.EXPLODE("Eating Disorders") | Exploded thesaurus term and major heading |
|  | OR | “eating disorder*” | Keyword |
|  | OR | “eating problem*” | Keyword |
|  | OR | anorexi* | Keyword |
|  | OR | bulimi* | Keyword |
|  | OR | "binge eat*" | Keyword |
|  | OR | “eating disorder not otherwise specified” | Keyword |
|  | OR | EDNOS | Keyword |
|  | OR | “unspecified feeding or eating disorder” | Keyword |
|  | OR | UFED | Keyword |
| ***Concept 2: Adverse life events*** | AND | MJMAINSUBJECT.EXACT.EXPLODE("Emotional Trauma") | Exploded major subject heading |
|  | OR | advers* | Keyword |
|  | OR | trauma* | Keyword |
|  | OR | “parental loss” | Keyword |
|  | OR | “witness* abuse*” | Keyword |
|  | OR | bereave* | Keyword |
|  | OR | “parent* divorce*” | Keyword |
|  | OR | “family problem*” | Keyword |
|  | OR | “early life stress*” | Keyword |
|  | OR | “cumulative advers*” | Keyword |
