# Supplementary material for: Family-related non-abuse adverse life experiences occurring for adults diagnosed with eating disorders: a systematic review
Source: J Eat Disord. 2020 Jul 22;8:36. doi: 10.1186/s40337-020-00311-6 (PMC7374817; doi:10.1186/s40337-020-00311-6)
Supplement: Supplementary file 2 — Additional file 2. Tabulation of results addressing research questions 2, 3 and 4. [file 40337_2020_311_MOESM2_ESM.docx]

**Additional file 2: Tabulation of results addressing research questions 2, 3 and 4**

**Table 2:** Data extraction of study findings in relation to rates, ED vs. control group comparisons, ED vs. PC group comparisons and ED subgroup comparisons for each form of family-related non-abuse ALEs

| Study | Non-abuse ALE subtype | Rates | | | | | | | | | | | | | | | | ED vs. control comparisons | ED vs. psychiatric control comparisons | ED subgroup comparisons |
| --- | --- | --- | --- | --- | --- | --- | --- | --- | --- | --- | --- | --- | --- | --- | --- | --- | --- | --- | --- | --- |
| 1. Adverse parenting style | | | | | | | | | | | | | | | | | | | | |
| (i) Calam et al. (1990) | Maternal care |  | | | | | | | | | | | | | | | | BNX < AN, BN/Hx AN and C (*p* < 0.02) |  |  |
|  | Maternal control |  | | | | | | | | | | | | | | | | AN = BN/Hx AN = BNX = C  (*p* > 0.05) |  |  |
|  | Paternal care |  | | | | | | | | | | | | | | | | BNX and BN/Hx AN < AN and C (*p* < 0.05) |  |  |
|  | Paternal control |  | | | | | | | | | | | | | | | | AN = BN/Hx AN = BNX = C (*p* > 0.05) |  |  |
| (ii) Connan et al. (2007) | Parental indifference |  | | | | | | | | | | | | | | | | AN and R-AN > C (*p* = 0.002) |  | AN = R-AN (n.s.) |
|  | Parental overcontrol |  | | | | | | | | | | | | | | | | AN and R-AN > C (*p* = 0.005) |  |  |
| (iii) Gonçalves et al. (2016) | Excessive parent control | BN  45.0% | | | | | C  30.0% | | | | | | PC  40.0% | | | | | BN = C (n.s.) | - |  |
|  | Parental non-involvement | BN  76.7% | | | | | C  43.3% | | | | | | PC  51.7% | | | | | BN > C (*p* = 0.01) | BN = PC (n.s.) |  |
|  | Parental high expectations | BN  76.7% | | | | | C  38.3% | | | | | | PC  53.3% | | | | | BN > C (*p* < 0.001) | BN > PC (*p* = 0.04) |  |
|  | Parental criticism | BN  45.0% | | | | | C  15.0% | | | | | | PC  45.0% | | | | | BN > C (*p* = 0.02) | - |  |
|  | Maternal non-involvement | BN  48.3% | | | | | C  21.7% | | | | | | PC  31.7% | | | | | BN = C (n.s.) | BN = PC (n.s.) |  |
|  | Maternal over-involvement | BN  20.0% | | | | | C  0% | | | | | | PC  5.0% | | | | | - | BN = PC (n.s.) |  |
|  | Maternal high expectations | BN  68.3% | | | | | C  25.0% | | | | | | PC  41.7% | | | | | BN > C (*p* < 0.001) | BN > PC (*p* = 0.03) |  |
|  | Maternal criticism | BN  36.7% | | | | | C  6.7% | | | | | | PC  30.0% | | | | | BN > C (*p* = 0.009) | - |  |
|  | Father non-involvement | BN  58.3% | | | | | C  40.0% | | | | | | PC  53.3% | | | | | BN = C (n.s.) | - |  |
|  | Father high expectations | BN  68.3% | | | | | C  36.7% | | | | | | PC  38.3% | | | | | BN > C (*p* = 0.002) | BN > PC (*p* = 0.002) |  |
|  | Father criticism | BN  23.3% | | | | | C  8.3% | | | | | | PC  21.7% | | | | | BN > C (*p* = 0.03) | - |  |
| (iv) Lehoux & Howe (2007) | Maternal affection |  | | | | | | | | | | | | | | | | BN = sisters * |  |  |
|  | Maternal control |  | | | | | | | | | | | | | | | | BN > sisters (*p* < 0.001, as reported by sisters) |  |  |
|  | Paternal affection |  | | | | | | | | | | | | | | | | BN = sisters * |  |  |
|  | Paternal control |  | | | | | | | | | | | | | | | | BN > sisters (*p* < 0.001, as reported by sisters) |  |  |
| (v) Mangweth et al. (1997) | Quality of relationship with father |  | | | | | | | | | | | | | | | | ED < C * |  |  |
|  | Tenderness felt from mother |  | | | | | | | | | | | | | | | | ED < C * |  |  |
| (vi) Monteleone et al. (2019) | Maternal care |  | | | | | | | | | | | | | | | | C > ANR and BP (*p* < 0.01) |  | ANR = BP (n.s.) |
|  | Maternal control |  | | | | | | | | | | | | | | | | ANR and BP > C (*p* < 0.01) |  | ANR = BP (n.s.) |
|  | Paternal care |  | | | | | | | | | | | | | | | | C > ANR and BP (*p* < 0.01) |  | ANR = BP (n.s.) |
|  | Paternal control |  | | | | | | | | | | | | | | | | ANR and BP > C (*p* < 0.01) |  | ANR = BP (n.s.) |
| (vii) Pike et al. (2008) | Maternal problem parenting |  | | | | | | | | | | | | | | | | AN and PC > C (*p* = 0.002) | AN = PC (n.s.) |  |
|  | Paternal problem parenting |  | | | | | | | | | | | | | | | | AN and PC > C (*p* < 0.001) | AN = PC (n.s.) |  |
|  | High parental demands |  | | | | | | | | | | | | | | | | AN and PC > C (*p* = 0.002) | AN > PC (*p* < 0.01) |  |
| (viii) Schmidt et al. (1993) | Parental indifference | RAN  13% | | | | BAN  17% | | | BN/HAN  30% | | | | | BN  38% | | | |  |  | BN > RAN, BAN, BN/HAN (*p* = 0.004) |
|  | Parental over-control | RAN  19% | | | | BAN  22% | | | BN/HAN  24% | | | | | BN  43% | | | |  |  | BN > RAN, BAN, BN/HAN (*p* = 0.009) |
|  | Parental under-control | RAN  17% | | | | BAN  17% | | | BN/HAN  16% | | | | | BN  19% | | | |  |  | BN = RAN = BAN = BN/HAN (n.s.) |
| (ix) Striegel-Moore et al. (2005) | Maternal problem parenting |  | | | |  | | |  | | | | |  | | | | BED + PC > control (p = 0.007) |  |  |
|  | Paternal problem parenting |  | | | |  | | |  | | | | |  | | | | BED = PC = control (p > 0.01) |  |  |
|  | High parental demands |  | | | |  | | |  | | | | |  | | | |  | BED > PC (p < 0.0001) |  |
| (x) Swanson et al. (2010) | Maternal care |  | | | | | | | | | | | | | | | | AN < C (*p* < 0.05) |  |  |
|  | Paternal care |  | | | | | | | | | | | | | | | | AN < C (*p* < 0.05) |  |  |
|  | Maternal control |  | | | | | | | | | | | | | | | | AN = C (n.s.) |  |  |
|  | Paternal control |  | | | | | | | | | | | | | | | | AN = C (n.s.) |  |  |
| (xi) Wade et al. (2007) | Parental expectations |  | | | | | | | | | | | | | | | | D1:  MD > C (*p* < 0.05)  AN = BN = C (n.s.)  D2a:  AN = TC (n.s.)  BN = TC (n.s.)  MD = TC (n.s.) | D2b:  BN > MD (*p* < 0.05)  AN = MD (n.s.) |  |
|  | Parental criticism |  | | | | | | | | | | | | | | | | D1:  BN and MD > C (*p* < 0.05)  AN = C (n.s.)    D2a:  AN = TC (n.s.)  BN = TC (n.s.)  MD > C (*p* < 0.05) | D2b:  AN and BN > MD (*p* < 0.05) |  |
|  | Maternal care |  | | | | | | | | | | | | | | | | D1:  AN = BN = MD = C (n.s.)  D2a:  AN = TC (n.s.)  BN = TC (n.s.)  MD = TC (n.s.) | D2b:  AN = BN = MD (n.s.) |  |
|  | Paternal care |  | | | | | | | | | | | | | | | | D1:  AN = BN = MD = C (n.s.)  D2a:  AN = TC (n.s.)  BN > TC (*p <* 0.05)  MD = TC (n.s.) | D2b:  AN = BN = MD (n.s.) |  |
|  | Maternal over-protectiveness |  | | | | | | | | | | | | | | | | D1:  AN = BN = MD = C (n.s.)  D2a:  AN = TC (n.s.)  BN = TC (n.s.)  MD = TC (n.s.) | D2b:  AN = BN = MD (n.s.) |  |
|  | Paternal over-protectiveness |  | | | | | | | | | | | | | | | | D1:  AN > C (*p* < 0.05)  BN = MD = C (n.s.)  D2a:  AN > TC (*p* < 0.05)  BN = TC (n.s.)  MD = TC (n.s.) | D2b:  AN = BN = MD (n.s.) |  |
| (xii) Webster & Palmer (2000) | Parental indifference | BN  34% | | AN  4% | | | AN+BN  40% | | | | DEP  40% | | | C  13% | | | | BN > C (*p* < 0.05)  AN+BN > C (*p* < 0.01)  AN = C (n.s.)  DEP > C (*p* < 0.01) | DEP > AN (*p <* 0.05)  DEP = BN (n.s.)  DEP = AN+BN (n.s.) | BN > AN (*p <* 0.05) |
|  | Lax control | BN  28% | | AN  7% | | | AN+BN  25% | | | | DEP  18% | | | C  20% | | | | BN = C (n.s.)  AN+BN = C (n.s.)  AN = C (n.s.)  DEP = C (n.s.) | DEP = AN (n.s.)  DEP = BN (n.s.)  DEP = AN+BN (n.s.) | BN = AN (n.s.) |
|  | High control | BN  13% | | AN  4% | | | AN+BN  20% | | | | DEP  28% | | | C  8% | | | | BN = C (n.s.)  AN+BN = C (n.s.)  AN = C (n.s.)  DEP > C (*p* < 0.05) | DEP = AN (n.s.)  DEP = BN (n.s.)  DEP = AN+BN (n.s.) | BN = AN (n.s.) |
|  | Lack of care | BN  47% | | AN  11% | | | AN+BN  50% | | | | DEP  43% | | | C  23% | | | | BN > C (*p* < 0.05)  AN+BN > C (*p* < 0.05)  AN = C (n.s.)  DEP = C (n.s.) | DEP > AN (*p <* 0.05)  DEP = BN (n.s.)  DEP = AN+BN (n.s.) | BN > AN (*p <* 0.05) |
| 1. Family disharmony | | | | | | | | | | | | | | | | | | | | |
| (i) Boumann & Yates (1994) | Parental divorce | BN  28% | | | | | | | C  4% | | | | | | | | | BN > C (OR = 4.9) |  |  |
| (ii) Degortes et al. (2014) | Family conflicts | BED  45.8% | | | | | | | BN  56.1% | | | | | | | | |  |  | BED = BN (n.s.) |
| (iii) Gonçalves et al. (2016) | Unresolved disagreements | BN  55% | | | | | C  8.3% | | | | | | PC  23.3% | | | | | BN > C (*p* = 0.001) | BN > C (*p* = 0.003) |  |
| (iv) Machado et al. (2014) | Parental arguments | AN  33.7% | | | | | | | C  20.9% | | | | | | | | | AN = C (n.s.) |  |  |
|  | Participant arguments with parents | AN  19.8%  AN  17.6% | | | | | | | C  10.5%  PC  33.8% | | | | | | | | | AN = C (n.s.) | AN = PC (n.s.) |  |
|  | Arguments within home | AN  34.9% | | | | | | | C  19.8% | | | | | | | | | AN = C (n.s.) |  |  |
|  | Unresolved/ unaddressed family disagreements | AN  52.3%  AN  51.5% | | | | | | | C  10.5%  PC  33.8% | | | | | | | | | AN > C (*p <* 0.001) | Trend towards AN > PC (*p =* 0.018) |  |
|  | Sibling rivalry | AN  17.4%  AN  16.2% | | | | | | | C  5.8%  PC  7.4% | | | | | | | | | AN = C (n.s.) | AN = PC (n.s.) |  |
| (v) Mangweth et al. (1997) | Witnessing parental violence |  | | | | | | | | | | | | | | | | ED > C * |  |  |
|  | Perceived parental marital dissatisfaction |  | | | | | | | | | | | | | | | | ED > C * |  |  |
| (vi) Noordenbos et al. (2002) | Problems with family | ED = 38% | | | | | | | | | | | | | | | |  |  |  |
|  | Problems with mother | ED = 45% | | | | | | | | | | | | | | | |  |  |  |
|  | Problems with father | ED = 28% | | | | | | | | | | | | | | | |  |  |  |
| (vii) Pike et al. (2008) | Family discord |  | | | | | | | | | | | | | | | | AN > C (p < 0.01) | AN > PC (p < 0.01) |  |
| (viii) Schmidt et al. (1993) | Family discord (i.e. tension) | RAN  47% | | | | BAN  39% | | | BN/HAN  59% | | | | | BN  64% | | | |  |  | Trend toward BN > RAN, BAN and BN/HAN (*p* = 0.64) |
| (ix) Striegel-Moore et al. (2005) | Family discord |  | | | |  | | |  | | | | |  | | | |  | BED > PC (p < 0.01) |  |
| (x) Wade et al. (2007) | Parental conflict |  | | | | | | | | | | | | | | | | D1:  Trend towards BN > C (*p* = 0.07)  AN = C (n.s.)  MD > C (*p* < 0.05)  D2a:  BN > TC (*p* < 0.05)  AN = TC (n.s.)  MD = TC (n.s.)  D2b:  AN and MD > C (*p* < 0.05)  BN = C (n.s.) | D2b:  AN = BN = MD (n.s.) |  |
| (xi) Webster & Palmer (2002) | Family discord (e.g. arguments and tension) | BN  53% | | AN  32% | | | AN/BN  50% | | | | DEP  50% | | | C  23% | | | | BN and DEP > C (*p <* 0.01)  AN/BN > C (*p <* 0.05)  AN = C (n.s.) | BN and AN/BN and AN = DEP (n.s.) | AN = BN (n.s.) |
| 1. Loss of family member, relative or someone close | | | | | | | | | | | | | | | | | | | | |
| (i) Dalle Grave et al. (1996) | Loss of a family member | BED  17% | | ANR  7% | | | | ANB  0% | | | BN  6% | | | | | OB  0% | |  |  |  |
| (ii) Dalle Grave et al. (1996) | Loss of a family member | BED  17% | ANR  7% | | | | ANB  9% | | BN  4% | | | SCZ  0% | | | | | C  2.6% |  |  |  |
| (iii) Degortes et al. (2014) | Bereavement of relative/ close friend | BED  26.2% | | | | | | | BN  14% | | | | | | | | |  |  | Trend towards BED > BN (*p* = 0.026) |
| (iv) Manwaring et al. (2006) | Loss of someone close |  | | | | | | | | | | | | | | | |  |  | Binge-first BED > diet-first BED (*p* = 0.003) |
| (v) Pike et al.  (2006) | Loss of someone close | BED  19.1%  BED  19.6% | | | | | | | C  5.6%  PC  9.3% | | | | | | | | | BED > C (p < 0.001) | BED = PC (n.s.) |  |
| (vi) Pike et al. (2008) | Parental absence or death |  | | | | | | | | | | | | | | | | AN = PC = C (n.s.) | AN = PC (n.s.) |  |
|  | Death of a relative, partner or close friend |  | | | | | | | | | | | | | | | | AN = PC = C (n.s.) | AN = PC (n.s.) |  |
| (vii) Reid et al. (2019) | Death of close family member | ED  100% | | | | | | | | | | | | | | | |  |  |  |
| (viii) Schmidt et al. (1993) | Parental death | RAN  3% | | | | BAN  4% | | | BN/HAN  3% | | | | | BN  6% | | | |  |  |  |
| (ix) Tagay et al. (2014) | Death of close person or family member | AN  49% | | | | | | | BN  50% | | | | | | | | |  |  | AN = BN (n.s.) |
| 1. Familial mental health issues | | | | | | | | | | | | | | | | | | | | |
| (i) Arthur-Cameselle et al. (2017) | Family dysfunction (i.e. parental psychological instability, substance abuse) | A  42% | | | | | | | NA  59% | | | | | | | | |  |  |  |
|  | Family members with ED or obesity | A  25% | | | | | | | NA  53% | | | | | | | | |  |  |  |
| (ii) Boumann & Yates (1994) | Parental psychiatric disorder | BN  44% | | | | | | | C  14% | | | | | | | | | BN > C (*p* < 0.05) |  |  |
|  | Parental MD | BN  28% | | | | | | | C  10% | | | | | | | | | BN > C (*p* < 0.05) |  |  |
|  | Parental personality disorder | BN  28% | | | | | | | C  6% | | | | | | | | | BN > C (*p* < 0.05) |  |  |
|  | Parental alcoholism | BN  10% | | | | | | | C  4% | | | | | | | | | BN = C (n.s.) |  |  |
| (iii) Cuijpers et al. (1999) | EDs in ACOAs and non-ACOAs | non-ACOA males  0.1%  non-ACOA females  1.2% | | | | | | | ACOA males  2.0%  ACOA females  2.1% | | | | | | | | | ACOA males > Non-ACOA males (*p* < 0.05)  ACOA females = non-ACOA females (n.s.) |  |  |
| (iv) Gonçalves et al. (2016) | Family depression | BN  61.7% | | | | | C  31.7% | | | | | | PC  60.0% | | | | | BN > C (*p* = 0.02) | - |  |
|  | Parental depression | BN  46.7% | | | | | C  31.7% | | | | | | PC  51.7% | | | | | BN = C (n.s.) | - |  |
|  | Family alcohol abuse | BN  35.0% | | | | | C  20.0% | | | | | | PC  35.0% | | | | | BN = C (n.s.) | - |  |
|  | Family drug abuse | BN  23.3% | | | | | C  10.0% | | | | | | PC  13.3% | | | | | BN = C (n.s.) | - |  |
|  | Family obsessive compulsive disorder | BN  18.3% | | | | | C  0% | | | | | | PC  3.3% | | | | | - | BN = PC (n.s.) |  |
|  | Premorbid family depression | BN  36.7% | | | | | C  23.3% | | | | | | PC  38.3% | | | | | BN = C (n.s.) | - |  |
|  | Premorbid family drug abuse | BN  16.7% | | | | | C  5.0% | | | | | | PC  10.0% | | | | | BN = C (n.s.) | - |  |
|  | Family member with ED (current) | BN  21.7% | | | | | C  3.3% | | | | | | PC  1.7% | | | | | - | BN > PC (*p* = 0.004) |  |
|  | Family member with ED (ever) | BN  35.0% | | | | | C  5.0% | | | | | | PC  5.0% | | | | | BN > C (*p* = 0.036) | - |  |
| (v) Machado et al. (2014) | Family history of AN or BN ever | AN  26.7%  AN  30.9% | | | | | | | C  9.3%  PC  4.4% | | | | | | | | | AN > C (*p* = 0.008) | AN > PC (*p* = 0.002) |  |
|  | Family alcoholism | AN  27.9% | | | | | | | C  16.3% | | | | | | | | | AN = C (n.s.) |  |  |
|  | Parental alcoholism | AN  12.8%  AN  13.2% | | | | | | | C  4.7%  PC  26.5% | | | | | | | | | AN = C (n.s.) | AN = PC (n.s.) |  |
| (vi) Pike et al. (2008) | Family history of AN |  | | | | | | | | | | | | | | | | AN = PC = C (n.s.) | AN = PC (n.s.) |  |
|  | Family history of BN |  | | | | | | | | | | | | | | | | AN = PC = C (n.s.) | AN = PC (n.s.) |  |
|  | Parental mood and substance disorders |  | | | | | | | | | | | | | | | | AN = PC = C (n.s.)  AN/PC > C (*p* < 0.01) | AN = PC (n.s.) |  |
| (vii) Schmidt et al. (1993) | Maternal mental health issue | RAN  28% | | | | BAN  22% | | | BN/HAN  35% | | | | | BN  27% | | | |  |  | RAN = BAN = BN/HAN = BN (n.s.) |
|  | Paternal mental health issue | RAN  19% | | | | BAN  26% | | | BN/HAN  14% | | | | | BN  21% | | | |  |  | RAN = BAN = BN/HAN = BN (n.s.) |
| (viii) Striegel-Moore et al. (2005) | Parental mood or substance disorder |  | | | |  | | |  | | | | |  | | | | BED + PC > control (p < 0.0001) |  |  |
| 1. Family comments about eating, or weight, shape and appearance | | | | | | | | | | | | | | | | | | | | |
| (i) Arthur-Cameselle et al. (2017) | Family negative comments about body or eating | A  0% | | | | | | | NA  53% | | | | | | | | |  |  |  |
| (ii) Duarte & Pinto-Gouveia (2017) | Critical comments about weight, shape or appearance | BED = 35.1%** | | | | | | | | | | | | | | | |  |  |  |
| (iii) Gonçalves et al. (2016) | Family repeated comments about weight | BN  76.7% | | | | | C  31.7% | | | | | | PC  40.0% | | | | | BN > C (*p* = 0.009) | BN > C (*p* = 0.01) |  |
|  | Family repeated comments about eating | BN  58.3% | | | | | C  31.7% | | | | | | PC  45.0% | | | | | BN = C (n.s.) | - |  |
| (iv) Machado et al. (2014) | Critical comments by family about shape or weight | AN  53.5%  AN  60.3% | | | | | | | C  37.2%  PC  45.6% | | | | | | | | | AN = C (n.s.) | AN = PC (n.s.) |  |
|  | Parents repeated comments about eating | AN  55.8%  AN  60.3% | | | | | | | C  30.2%  PC  36.8% | | | | | | | | | Trend towards AN > C (*p* = 0.012) | AN = PC (n.s.) |  |
| (v) Sweetingham & Waller (2008) | Teasing about appearance | ED = 14.1% | | | | | | | | | | | | | | | |  |  |  |
| (vi) Wade et al. (2007) | Parental comments about weight |  | | | | | | | | | | | | | | | | D1:  AN and BN > C (*p* < 0.05)  MD = C (n.s.)  D2a:  AN > TC (*p* < 0.05)  BN and MD = TC (n.s.) | D2b:  BN > MD and C  (*p* < 0.05) |  |
|  | Parental comments about amount eaten |  | | | | | | | | | | | | | | | | D1:  AN, BN and MD > C  (*p* < 0.05)  D2a:  AN and MD> TC (*p* < 0.05)  BN = TC (n.s.) | D2b:  AN, BN > MD and C  (*p* < 0.05) |  |
| 1. Disruptions in family structure | | | | | | | | | | | | | | | | | | | | |
| (i) Degortes et al. (2014) | Separation of family members | BED  20.6% | | | | | | | | BN  7.5% | | | | | | | |  |  | BED > BN (*p* = 0.005) |
| (ii) Gonçalves et al. (2016) | Change of parental figure | BN  18.3% | | | | | C  6.7% | | | | | | PC  13.3% | | | | | BN = C (n.s.) | BN = PC (n.s.) |  |
| (iii) Pike et al. (2006) | Member leaving or joining family structure | BED  28.6%  BED  32.4% | | | | | | | | C  11.2%  PC  14.5% | | | | | | | | BED > C (*p* < 0.001) | BED > PC (*p =* 0.005) |  |
| (iv) Pike et al. (2008) | Member leaving or joining family structure |  | | | | | | | |  | | | | | | | | AN = C (n.s.) | AN = PC (n.s.) |  |
| (v) Schmidt et al. (1993) | Adoption | RAN  0% | | | BAN  0% | | | | | BN/HAN  5% | | | | | BN  3% | | |  |  |  |
|  | Parental separation | RAN  17% | | | BAN  13% | | | | | BN/HAN  22% | | | | | BN  27% | | |  |  |  |
| (vi) Striegel-Moore et al. (2005) | Separation from parents |  | | | | | | | | | | | | | | | | BED + PC > control (p < 0.0001) |  |  |

BNX = Bulimia with no history of Anorexia; AN = anorexia nervosa; BN/Hx AN = BN with a history of AN; C = control; R-AN = Recovered AN; BN = bulimia nervosa; ED = Eating disorder; ANR = AN restrictive subtype; BP = bingeing-purging; PC = psychiatric controls; RAN = restrictive AN; BAN = Bulimic AN; BN/HAN = BN with a history of anorexia; BED = binge eating disorder; MD = major depression; TC= twin control; AN+BN = mixed anorexia and bulimia; DEP = depression; ACOA = adult children of alcoholics; Non-ACOA = non adult children of alcoholics; AN/PC = mixed AN and PC group ANB= Anorexia bulimic subtype; OB = obesity; SCZ = schizophrenia; A = athlete; NA = non-athlete

*n.s.* = non-significant; * = *p* values not reported; ** also includes comments made by peers; *OR* = odds ratio; D1= Design 1: Diagnostic group comparisons; D2a= Design 2a: Monozygotic twin pairwise comparisons; D2b= Design 2b: Monozygotic twin case control; Hyphen sign (-) indicates that data failed to meet statistical assumptions and therefore data were not analysed statistically.
